# The Performance of an Oral Microbiome Biomarker Panel in Predicting Oral Cavity and Oropharyngeal Cancers

**DOI:** 10.3389/fcimb.2018.00267

**Published:** 2018-08-03

**Authors:** Yenkai Lim, Naoki Fukuma, Makrina Totsika, Liz Kenny, Mark Morrison, Chamindie Punyadeera

**Affiliations:** ^1^The School of Biomedical Sciences, Institute of Health and Biomedical Innovation, Queensland University of Technology, Brisbane, QLD, Australia; ^2^Translational Research Institute, Brisbane, QLD, Australia; ^3^The Department of Life and Food Science, Obihiro University of Agriculture and Veterinary Medicine, Obihiro, Japan; ^4^The School of Medicine, University of Queensland, Royal Brisbane and Women's Hospital, Brisbane, QLD, Australia; ^5^Translational Research Institute, University of Queensland Diamantina Institute, University of Queensland, Brisbane, QLD, Australia

**Keywords:** oral cancer, oral rinse, saliva, biomarker, oral microbiome

## Abstract

The oral microbiome can play a role in the instigation and progression of oral diseases that can manifest into other systemic conditions. These associations encourage the exploration of oral dysbiosis leading to the pathogenesis of cancers. In this study, oral rinse was used to characterize the oral microbiome fluctuation associated with oral cavity cancer (OCC) and oropharyngeal cancers (OPC). The study cohort consists of normal healthy controls (*n* = 10, between 20 and 30 years of age; *n* = 10, above 50 years of age), high-risk individuals (*n* = 11, above 50 years of age with bad oral hygiene and/or oral diseases) and OCC and OPC patients (*n* = 31, HPV-positive; *n* = 21, HPV-negative). Oral rinse samples were analyzed using 16S rRNA gene amplicon sequencing on the MiSeq platform. Kruskal–Wallis rank test was used to identify genera associated with OCC and OPC. A logistic regression analysis was carried out to determine the performance of these genera as a biomarker panel to predict OCC and OPC. In addition, a two-fold cross-validation with a bootstrap procedure was carried out in R to investigate how well the panel would perform in an emulated clinical scenario. Our data indicate that the oral microbiome is able to predict the presence of OCC and OPC with sensitivity and specificity of 100 and 90%, respectively. With further validation, the panel could potentially be implemented into clinical diagnostic and prognostic workflows for OCC and OPC.

## Introduction

Head and neck cancers (HNC) describe a broad category of aggressive heterogeneous tumor types arising from the upper aerodigestive tract (Lim et al., 2016b). Globally, HNC were responsible for 529,500 incident cases and 292,300 deaths in 2012, accounting for ~3.8% of all cancer cases and 3.6% of cancer related deaths (Shield et al., 2017). Oral cavity cancers (OCC) and oropharyngeal cancers (OPC) constitute the majority of HNC with 300,000 men and 130,000 women being diagnosed annually (Adilbay et al., 2018). The number of newly diagnosed HNC patients is predicted to increase by 62% by 2035, equating to 856,000 new cases due to demographic changes (Shield et al., 2017). While the risk factors for OCC in developing countries are mainly excessive tobacco and alcohol consumption, human papillomavirus (HPV) infection is a rising etiological factor for OPC in developed countries (Kulasinghe, 2015).

Early recognition of the symptoms and signs, as well as prompt diagnosis of OCC and OPC is vital for patient survival. However, due to the lack of early screening/diagnostic tools, these cancer types are often diagnosed at advanced stages, resulting in poor survival outcomes (5-year mortality rate of 40–50%) (Lam et al., 2007; Kulasinghe, 2015; Lim et al., 2016a). To improve patient survival, saliva diagnostics have played a central role in the discovery of biomarkers for OCC and OPC early detection. This is due to the fact that most biomarkers present in blood and urine can also be detected in a sample of saliva (Malamud, 2011). A recent study by Wang et al. demonstrated the feasibility of detecting tumor DNA in saliva from OCC and OPC patients with a sensitivity of 100% in contrast to 80% sensitivity when plasma samples were used from the same patients (Wang et al., 2015). These findings substantiate saliva as an ideal diagnostic medium to detect OCC and OPC. Nevertheless, none of these host biomarkers were able to successfully translate into the clinical setting due to the high amount of biological variation between individuals (Lim et al., 2016b, 2017a).

In recent years, the human oral microbiome has emerged as a new potential biomarker reservoir for OCC and OPC (Lim et al., 2017a,b). The oral microbiome, by definition, is the collective genomes of microorganisms that reside in the oral cavity. Bacteria are the primary cause of oral diseases that affect other systemic conditions such as cardiovascular diseases, adverse pregnancy outcomes, diabetes mellitus and respiratory diseases (Lim et al., 2017a). These associations encourage the exploration of oral dysbiosis and the pathogenesis of OCC and OPC due to their close proximity. For example, the association between chronic periodontitis and OCC and OPC could be measured objectively based on periodontitis history (Tezal et al., 2005). Furthermore, the periodontal pocket consists of stratified squamous epithelium and is continuously undergoing epithelial proliferation, migration, rete-ridge formation and ulcerations, providing an ideal site for HPV infection and persistence (Tezal et al., 2009). It is well-known that persistent HPV infection leads to the development of OPC (Chai et al., 2015). The oral cavity has the largest core of commonly shared microbes among unrelated individuals (Zaura et al., 2014). The incorporation of the oral microbiome with salivary tumor biomarkers may thus help overcome the challenge of molecular diagnosis for OCC and OPC due to human biological variation (Lim et al., 2017a).

Based on recent studies that describe changes in the oral microbiome between healthy individuals and OCC and/or OPC patients, it was concluded that prevalent bacterial groups in cancer are associated with tumor development and progression (Pushalkar et al., 2012; Schmidt et al., 2014; Guerrero-Preston et al., 2016; Wang et al., 2017; Wolf et al., 2017). In addition, these bacterial groups could also be used as potential biomarkers for cancer detection, prognosis, and monitoring. However, the use of these prevalent bacterial groups as OCC/OPC biomarkers remains to be demonstrated in a clinical setting. Furthermore, these data report on the abundance changes of specific bacterial species/phylo-types as individual biomarkers. The oral microbiome is an intricate entity, and a reflection of the complex interactions between all members of the community (Lim et al., 2017a). Due to the obligatory dependencies of oral bacteria for growth and survival, we feel that further analyses of these microbial fluctuations as a biomarker panel in a clinical setting is warranted.

We hypothesize that the differences between the oral microbiomes of OCC and OPC patients and healthy individuals are reflected in oral rinse samples, and detectable via 16S rRNA gene amplicon sequencing. These differences could then be associated to the pathogenesis of cancers, and used as a biomarker panel to predict OCC and OPC with high diagnostic accuracy in an emulated clinical setting. To that end, our study objectives were three-fold: (i) to characterize microbiome differences in oral rinse samples from normal healthy controls and OCC and OPC patients; (ii) to develop a robust oral microbiome biomarker panel to predict OCC and OPC based on microbial fluctuations and; (iii) to evaluate the oral microbiome biomarker panel in an emulated clinical setting.

## Materials and methods

### Study cohort and sample collection

This study was approved by the Queensland University of Technology and University of Queensland Medical Ethical Institutional Boards (HREC no.: 1400000617 and HREC no.: 2017000662, respectively) and the Royal Brisbane and Women's Hospital (HREC no.: HREC/12/QPAH/381) Ethics Review Board. Written informed consent was obtained from all participants and all methods in this study were performed in accordance with the relevant guidelines and regulations. We have recruited normal healthy individuals between the ages of 20–30 years (*n* = 10) and above 50 years (*n* = 10) from the general population who self-reported to be in good general health. Individuals with bad oral hygiene and/or oral diseases such as gingivitis or periodontitis (*n* = 10) above the age of 50 years were also recruited in the same manner as high-risk population (full-mouth clinical examinations were performed by certified dentists). Newly diagnosed OCC and OPC patients were recruited upon diagnosis and were treatment naïve. Immunohistochemical detection of the p16^INK16a^ protein was used as surrogate marker for HPV status. All participants were not on any local and/or systemic antibiotics prior to sample collection.

Participants were asked to refrain from eating and drinking for an hour prior to sample collection. Bottled water was provided for participants to rinse their mouth before sampling. Oral rinse samples were collected by asking participants to swish and gargle with 10 mL of 0.9% (w/v) saline solution (Baxter International Incorporate, Illinois, USA) for a minute and expectorate into a 50 mL sterile Falcon tube as previously published (Chai et al., 2016; Lim et al., 2017b; Sun et al., 2017). After collection, all samples were transported back to the laboratory on dry ice and stored at −80°C.

### Bacterial genomic (g)DNA extraction

Total volume of 1 mL oral rinse samples were subjected to bacterial gDNA extraction using Maxwell® 16 LEV blood DNA kit (Promega Corporation, Wisconsin, USA) with an adapted protocol as published previously (Lim et al., 2017b).

### 16S rRNA gene amplicon library preparation and sequencing

16S rRNA gene amplicons for sequencing by Illumina MiSeq system (Illumina Incorporate, California, USA) was prepared according to the manufacturers' protocol with gene-specific sequences targeting the V6–V8 hypervariable regions (primers 926F and 1392R) of the 16S rRNA gene (Shanahan et al., 2016). Q5® Hot Start High-Fidelity (New England Biolabs, Massachusetts, USA) polymerase enzyme was used for the amplicon and index PCR. The sequencing was performed at the Australian Centre for Ecogenomics (ACE, Brisbane, Australia).

### Quantitative insights into microbial ecology (QIIME)

Illumina sequenced raw reads were analyzed using QIIME version 1.9.1 (Caporaso et al., 2010b). Chimeric sequences were identified and removed via USEARCH 6.1 to avoid perceived diversity (Edgar, 2010; Haas et al., 2011). Data sequences were clustered into operational taxonomic units (OTUs) by PyNAST with a 97% sequence identity threshold against Greengenes (chimera-checked 16S rRNA gene) core set database version 13.8 (DeSantis et al., 2006; Caporaso et al., 2010a; McDonald et al., 2012). Low abundance OTUs (≤0.1% of total sequences) and any sequences that were not of bacterial or archaeal origin were removed from the analysis. Based on the sample with the lowest OTU counts, random sampling (without replacement) was used to account for different sequencing depths that may occur for each individual sample.

### Statistical analysis

Rarefaction curve of the observed OTUs against sequences per sample and Shannon index was calculated using QIIME. On Calypso (version 5.4), bubble plot, partial least squares regression-discriminant analysis (PLS-DA, non-supervised) and redundancy analysis (supervised) was used to visualize the microbial communities (genus-level), based on OTU frequencies within respective categories in the metadata (Zakrzewski et al., 2017). In addition, the Kruskal–Wallis rank test was used to identify genera associated with OCC and OPC. Carstensen's multivariate receiver operating characteristic (ROC) curve with “Epi” package was used in R to evaluate the diagnostic potential of the biomarker panel. In brief, cancer status and microbial abundance (genus-level) was used as outcome and explanatory variable, respectively in a multivariable logistic regression statistical model. A predicted score was then generated for each sample using the estimated regression model and different cut-off values of this predicted score were used to classify samples into patients or controls. The performance of the biomarker panel was also tested in an emulated clinical setting by performing two-fold cross-validation test. In this case, a single subsample (randomly selected) was retained as the validation data for the testing model, and the remaining samples were used as training data. A bootstrap procedure was also implemented to repeat the process for 20 times to include all possible combinations of predictive model available (Devijver, 1982; Geisser, 1993; Kohavi, 1995).

## Results

### Population characteristics

Extracted bacterial gDNA from oral rinse samples were subjected to 16S rRNA gene amplicon sequencing. Based on our data, the average length of reads is 500 bp and the OTUs were subsampled to 1,044 counts. After processing, 770,061 high quality sequences were obtained in this study, with an average of 9,278 sequences per sample. From these sequences, 6 known phyla and 28 genera were identified, and a total of 108 OTUs were detected at the 97% sequence identify threshold.

The mean age for young normal healthy controls (between 20 and 30 years of age) was 26 years, and consisted of 8 males and 2 females. The mean age for elderly normal healthy controls (above 50 years of age) was 61 years and consisted of 4 males and 6 females. The mean age for high-risk controls (individuals with bad oral hygiene and/or oral diseases above 50 years of age) was 59 years and consisted of 8 males and 3 females (Supplementary Table 1). The patient cohort consists of mostly males with OCC or OPC and mean age of 65 years. A summary of the demographic and clinical characteristics of our patient cohort is presented in Table 1.

**Table 1:** The demographic characteristics of the patient cohort (*n* = 52).

**Explanatory variables**	**Patients**	
	**HPV-negative**	**HPV-positive**
	**(*n* = 21)**	**(*n* = 31)**
**DEMOGRAPHICS**		
**Gender**		
Male	16	30
Female	5	1
**Age**		
<50	1	0
>50	20	31
**Race and ethnicity**		
Caucasian	21	31
Other	0	0
**Smoking**		
Non-smoker	2	8
Ex-smoker	16	20
Smoker	3	3
**Alcohol**		
Non-drinker	7	19
Drinker	14	12
**TUMOR CHARACTERISTICS**		
**AJCC TNM stage**		
Stage I	3	1
Stage II	3	1
Stage III	6	4
Stage IV	9	25
**Tumor anatomic site**		
Oral cavity	10	5
Oropharyngeal	11	26

### Oral microbial profiles from normal healthy controls and cancer patients

**Figure 1:**
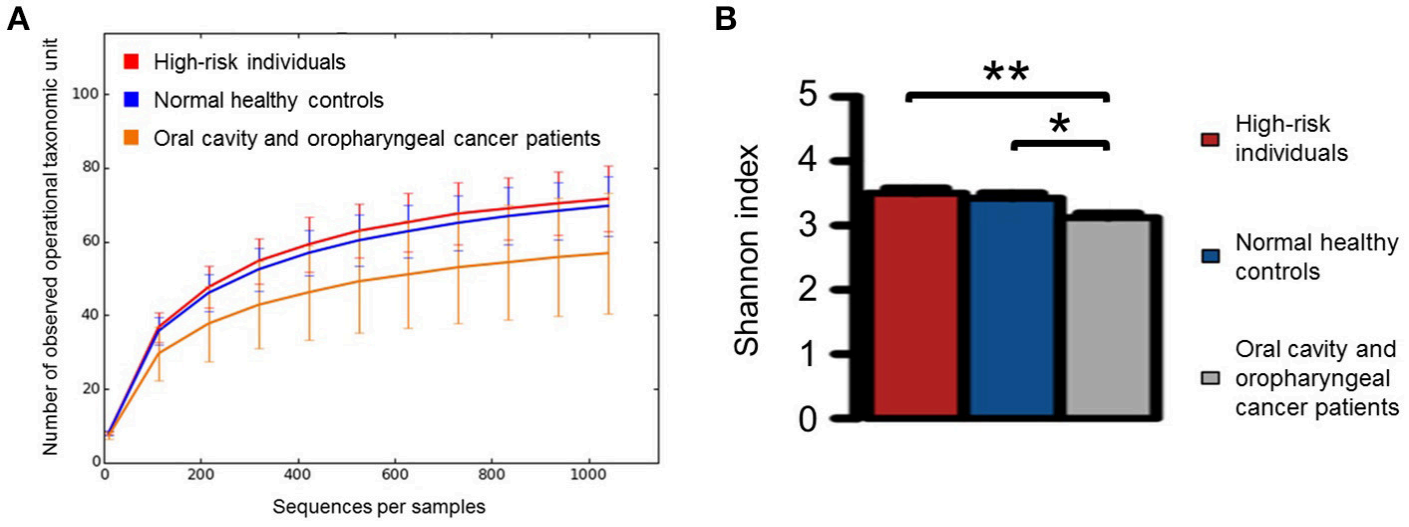
**(A)** Rarefaction curve of observed operational taxonomic unites against sequences per sample for normal healthy controls (*n* = 20), high-risk individuals (*n* = 11), and oral cavity and oropharyngeal cancer patients (*n* = 52) as well as **(B)** the comparison of Shannon index for each category using rank test. Significant differences are denoted with *P < 0.05 and **P < 0.01, respectively.

A rarefaction curve of observed OTUs against sequence per sample was plotted for normal healthy controls, high-risk individuals and OCC and OPC patients to determine the efficiency of the sequencing process. The Shannon index was also calculated for each cohort and compared using rank test, with the patient cohort having significantly lower species diversity compared with the normal healthy controls and high-risk individuals (Figures 1A,B).

**Figure 2:**
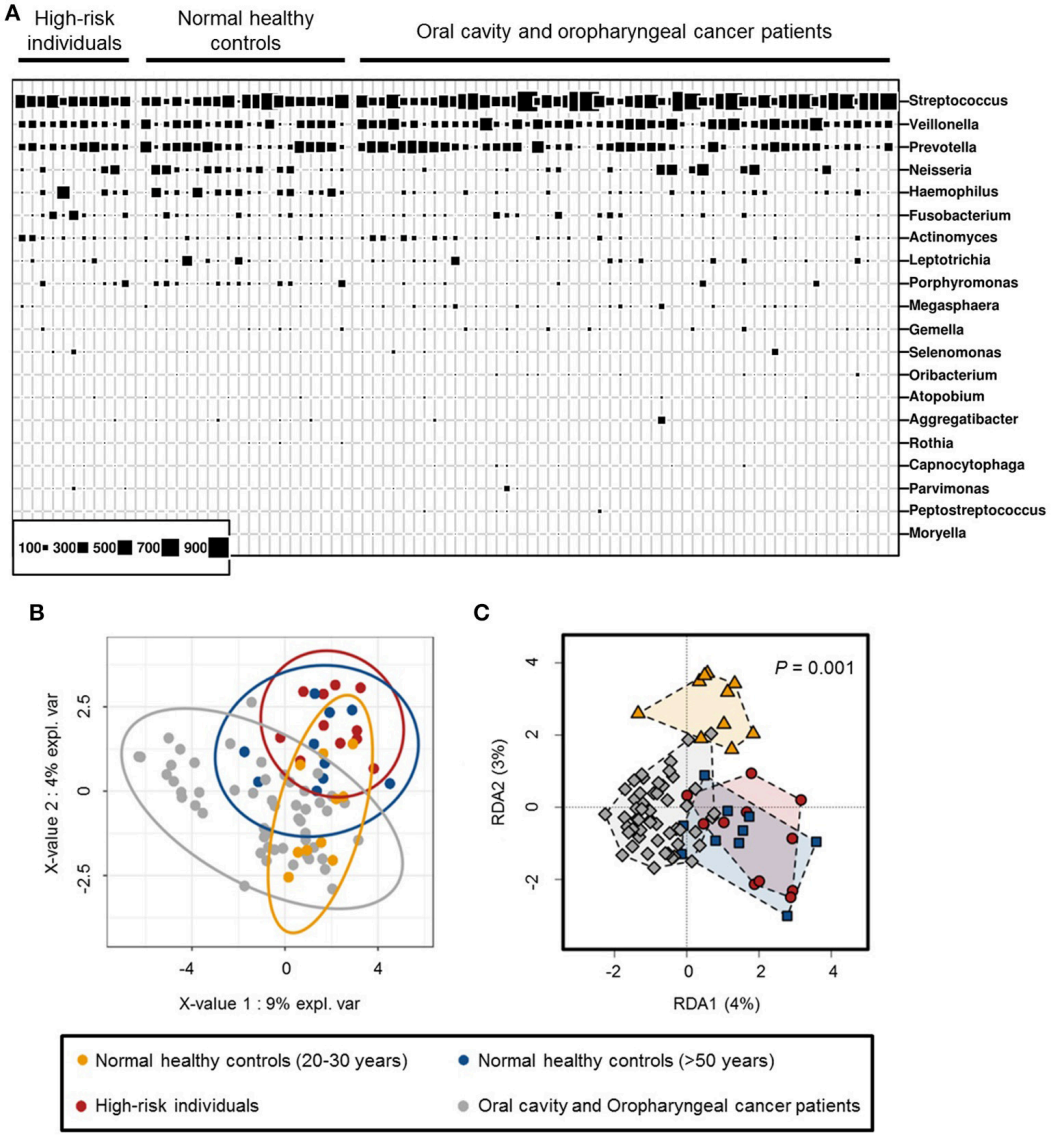
**(A)** Bubble plot of 20 most abundant bacterial genera detected in oral rinse samples of normal healthy controls (between 20 and 30 years of age, *n* = 10 and above 50 years of age, *n* = 10), high-risk individuals (*n* = 11) and oral cavity and oropharyngeal cancer patients (*n* = 52). **(B)** Partial least squares regression-discriminant analysis (PLS-DA) and **(C)** redundancy analysis of microbial communities (genus-level) in oral rinse samples, based on OTU frequencies within respective categories.

The taxonomic profiles of normal healthy controls, high-risk individuals and OCC and OPC patients were examined based on the proportion of bacterial sequences determined at genus-level (Figure 2A). The PLS-DA and redundancy plots show a clear distinct structure between normal healthy controls between the ages of 20–30 years and other categories (normal healthy controls above 50 years of age, high-risk individuals and OCC and OPC patients). While normal healthy controls above 50 years of age and high-risk individuals share similar features on both the 2-D plots, they are well separated from the cancer cohort (Figures 2B,C). Our results demonstrate that age and cancers may play a role in the shift of oral microbiome and are consistent with previous findings (Kang et al., 2006). Due to the association of age and oral microbiome, normal healthy controls between the ages of 20 and 30 years were removed from subsequent analysis to avoid inherent bias. According to Kruskal-Wallis rank test, *Rothia* (*P* < 0.0001), *Haemophilus* (*P* < 0.005), *Corynebacterium* (*P* < 0.01), *Paludibacter* (*P* < 0.01), *Porphyromonas* (*P* < 0.01), and *Capnocytophaga* (*P* < 0.05) are found in significantly lower abundance in oral rinse samples from OCC and OPC patients while *Oribacterium* (*P* < 0.05) is significantly higher. While *Actinomyces* (*P* < 0.05), *Parvimonas* (*P* < 0.05), *Selenomonas* (*P* < 0.05), and *Prevotella* (*P* < 0.05) have a significantly higher abundance in OCC compared with OPC; HPV has a positive correlation on the abundance of *Haemophilus* (*P* < 0.05) and *Gemella* (*P* = 0.06). Pathogenic and/or opportunistic bacteria such as *Actinomyces* (*P* < 0.01), *Actinobacillus* (*P* < 0.05), *Lautropia* (*P* < 0.05), *Fusobacterium* (*P* < 0.05) and *Aggregatibacter* (*P* < 0.05) are significantly more abundant in high-risk individuals (Supplementary Table 2).

### Oral microbiome biomarker panel and clinical significance

**Figure 3:**
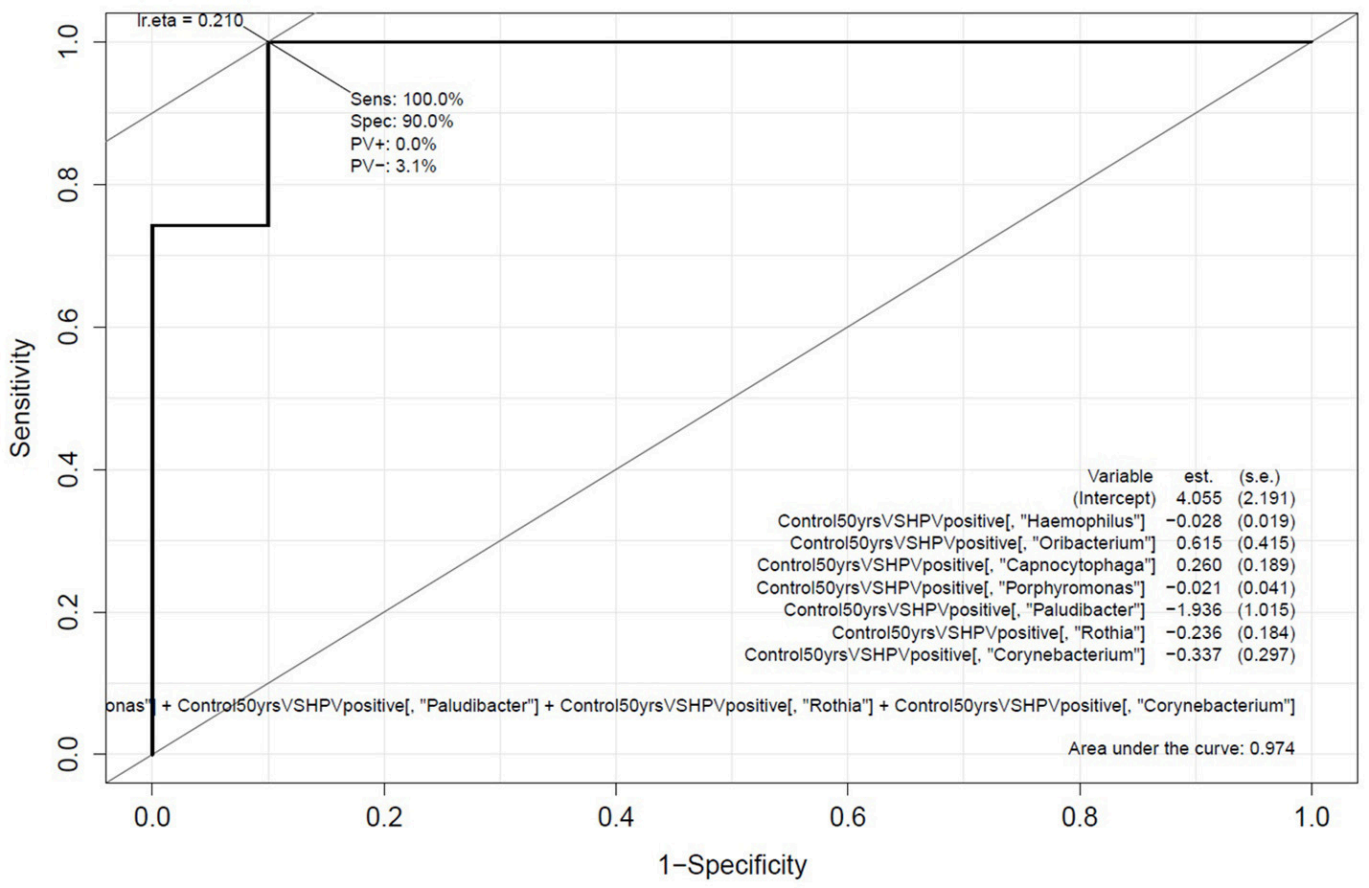
Performance of the oral microbiome panel in predicting oral cavity and oropharyngeal cancers. Carstensen's multivariate receiver-operating characteristics curve based on the abundance of *Rothia, Haemophilus, Corynebacterium, Paludibacter, Porphyromonas, Oribacterium*, and *Capnocytophaga*; comparing normal healthy controls (above 50 years of age, *n* = 10) with oral cavity and oropharyngeal cancer patients (*n* = 52).

Genera associated with OCC and OPC were selected as potential candidates to be included in the prediction panel. Hence, Carstensen's ROC curve for *Rothia, Haemophilus, Corynebacterium, Paludibacter, Porphyromonas, Oribacterium*, and *Capnocytophaga* were generated to investigate the panel's optimum performance based on the original samples that were used in building the model (Figure 3). With this approach, the panel has an area under curve (AUC) of 0.98 and sensitivity and specificity of 100 and 90%, respectively. The data were then processed using two-fold cross-validation and bootstrap to determine the performance of this panel in a “most likely scenario” with the intention of clinical translation. With the new enforced probability, the panel has an AUC of 0.82 and sensitivity and specificity of 90 and 61%, respectively (Supplementary Table 3). While the performance of the panel decreases, the AUC shows a prediction accuracy of 82%.

## Discussion

Oral dysbiosis is a frequent event in oral and oropharyngeal carcinogenesis (Tezal et al., 2005; Galvão-Moreira and da Cruz, 2016; Laprise et al., 2016; Moraes et al., 2016; Furquim et al., 2017). In this study, we utilized oral rinse as a platform for oral microbiome characterisation and described an oral microbiome panel (*Rothia, Haemophilus, Corynebacterium, Paludibacter, Porphyromonas, Oribacterium*, and *Capnocytophaga*) that can discriminate OCC and OPC patients from age-matched normal healthy individuals. Our findings concur with previous findings, in which the differences in microbial abundance and diversity may have a role in cancer initiation and progression (Lim et al., 2017a). To our knowledge, this is the first study that evaluates the performance of an oral microbiome panel to predict OCC and OPC in an emulated clinical setting.

Based on our data, age is a contributing factor for microbial differentiation in the oral environment. Due to the fact that OCC and OPC generally manifest in the sixth decade of life in addition to the mean age from our patient cohort (~65 years), individuals between the ages of 20 and 30 years were removed from the study to avoid the incorporation of any non-causal elements into the biomarker panel (Kang et al., 2006). While the microbial richness is generally consistent between all samples, OCC and OPC patients have a significantly lower diversity (Pushalkar et al., 2012; Schmidt et al., 2014; Guerrero-Preston et al., 2016). This may be due to the Warburg effect triggered by tumorigenesis, favoring the growth conditions of a subset of microbes (Cummins and Tangney, 2013). It is noted that while the majority of the genera have a lower abundance in cancers, *Oribacterium* was found to be significantly higher in abundance compared with normal healthy controls and high-risk individuals. In a recent study, Guerrero-Preston et al. also reported a higher *Oribacterium* abundance in OCC (Guerrero-Preston et al., 2016). This indicates that *Oribacterium* may play an important role in the pathogenesis of OCC and OPC.

HPV-positive OCC and OPC comprise a distinct molecular, clinical and pathologic disease entity that is likely causally associated with HPV infection (Gillison et al., 2000). In this study, we demonstrate that *Haemophilus* and *Gemella* have a positive correlation with HPV infection. A previous report described the increased abundance of *Leuconostoc* and *Gemella* in HPV-positive cancer samples (Guerrero-Preston et al., 2016). This differences may be due to the sample size variation as well as the demographics of the control cohort (in the previous study, individuals emitted to the same hospital with the cancer cohort for other diseases were used as controls provided that they have no history of cancer) (Guerrero-Preston et al., 2016). Nevertheless, these findings agree with previous studies on the impact of HPV on microbiome in cervical cancers (Mitra et al., 2015, 2016). It was also noted that certain genera were more abundant in OCC (*Actinomyces, Parvimonas, Selenomonas*, and *Prevotella*) compared with OPC. According to our previous study, there were no significant fluctuations in the microbiota composition between the oral and oropharyngeal regions, despite the distinct cellular, morphological and functional characteristics of these two regions (Lim et al., 2017b). Hence, we speculate that the microbiome differences may be directly influenced by the oral cavity tumor characteristics and/or secretomes.

Previous studies described oral diseases as a potential risk-factor for HNC (Tezal et al., 2005; Hasan and Palmer, 2014). In this study, we characterize the oral microbiome from individuals with gingivitis or periodontitis above the age of 50 years to determine if the oral microbiome could be used as a high-risk screening marker for OCC and OPC. Based on our data, the microbial composition from high-risk individuals bares a closer resemblance to normal healthy controls above the age of 50 years compared with OCC and OPC patients. This indicates that the oral microbiome differences detected in OCC and OPC patients may be cancer-specific, highlighting oral microbiome as an ideal biomarker candidate for cancer detection. However, due to the lack of dental records for the patient cohort, direct comparisons of oral health status could not be made. Future studies are warranted to compare the oral microbiome between high-risk individuals and cancer patients based on dental records to draw a more definite conclusion.

In previous studies, *Actinobacillus, Actinomyces, Aggregatibacter, Capnocytophaga, Fusobacterium, Oribacterium, Rothia, Haemophilus, Leptotrichia, Neisseria, Porphyromonas* and *Veillonella* were commonly found to be potential individual biomarker candidate for HNC due to the significant shift of abundance in cancer samples (Nagy et al., 1998; Mager et al., 2005; Schmidt et al., 2014; Guerrero-Preston et al., 2016; Wang et al., 2017; Wolf et al., 2017). Our investigation concurs with these findings. However, the compositions of *Aggregatibacter* and *Fusobacterium* between normal healthy controls and cancer patients are equivalent in our study. In addition to the genera mentioned, we also discovered a significant loss of abundance with *Paludibacter* and *Corynebacterium* in cancer patients that has not been reported previously. The oral microbiome panel was conceptualized by using genera that have a strong correlation with OCC and OPC via an inclusion-exclusion principle. The panel is able to predict OCC and OPC with a high sensitivity and specificity. Although the performance of the panel decreases when placed against a more realistic scenario, the panel's prediction accuracy was not significantly affected. This is a good clinical endpoint as the panel enables non-invasive OCC and OPC detection with a simple genera quantification test on oral rinse samples.

One of the limitations of this study is the modest sample size and the lack of validation system. However, despite the fact that inherent value often obtained with greater numbers, we were able to detect and report statistically significant differences between our cohorts of interest. In addition, our findings correlate with previously established studies that consist of similar or larger sample cohort (Schmidt et al., 2014; Guerrero-Preston et al., 2016; Wang et al., 2017; Wolf et al., 2017). These results provide support in conducting larger cohort validation in the future to substantiate the diagnostic value of the oral microbiome panel in OCC and OPC. In addition, to best of our knowledge, the oral microbiome association between active smokers and OCC and OPC patients has not been investigated in the past. In our experience, this is due to the difficulty in recruiting individuals above the age of 50 years who actively smoke while having no oral hygiene problems and underlying conditions (oral, lung, and cardiovascular diseases) that could potentially influence the oral microbiome (Weidlich et al., 2008; Aho et al., 2015; Menon et al., 2017; Yamashita and Takeshita, 2017). Studies have shown that smoking affects the oral microbiome; the oral microbiome panel will have to be tested on age-matched active smoking individuals in the future to avoid overrepresentation (Wu et al., 2016). A longitudinal study using the oral microbiome panel should also be carried out to monitor the oral microbiome changes before and after treatment with multiple follow-up studies. The rate of oral microbiome recovery measured using the panel may lead to personalized medicine.

Overall, the results of this study have shown that the oral microbiome panel of *Rothia, Haemophilus, Corynebacterium, Paludibacter, Porphyromonas, Oribacterium*, and *Capnocytophaga* were able to discriminate age-matched normal healthy controls from OCC and OPC patients with high accuracy. Furthermore, two advanced statistical models were used to demonstrate the clinical relevance of the panel. While previous studies focus on the oral microbiome differences between controls and cases, we were able to establish that the information is clinically useful. We hope the findings presented here will contribute to the translation of microbial profiling techniques into technologies that improve the diagnosis and treatment of OCC and OPC.

## Author contributions

YL collected samples, compiled all epidemiological data on all subjects, conducted all experiments, data interpretation, and wrote the manuscript. NF provided support on QIIME and Calypso and revised the manuscript critically for content. MT and MM contributed to the experimental design, laboratory facilities and technical support, data interpretation, and revised the manuscript critically for content. LK identified areas of tumor cellularity for all patients and conducted HPV assays on selected patients. CP participated in study concept and design, study coordination and revised the manuscript critically for content. All authors have read and approved the content of this manuscript.

### Conflict of interest statement

The authors declare that the research was conducted in the absence of any commercial or financial relationships that could be construed as a potential conflict of interest.

## Abbreviations

AUC: Area under curve
gDNA: Genomic DNA
HNC: Head and neck cancers
HPV: Human papillomavirus
OCC: Oral cavity cancer
OPC: Oropharyngeal cancer
OTU: Operational taxonomic unit
PLS-DA: Partial least squares–discriminant analysis
QIIME: Quantitative insights into microbial ecology
ROC: Receiver operating characteristic.
