# Supplementary material for: The Performance of an Oral Microbiome Biomarker Panel in Predicting Oral Cavity and Oropharyngeal Cancers
Source: Front Cell Infect Microbiol. 2018 Aug 3;8:267. doi: 10.3389/fcimb.2018.00267 (PMC6085444; doi:10.3389/fcimb.2018.00267)
Supplement: Supplementary file 1 [file Table_1.DOCX]

**Supplementary Table 1. Recruitment criteria and demography for normal healthy control and high-risk individual.**

**(a) Normal healthy control**

1. Must have a good general state of health.
2. No fever or signs and symptoms suggesting active infection/illness on the day of saliva donation.
3. No mouth ulcers, inflammation of the gums, halitosis, any mouth infection, dry mouth or sensitive teeth on the day of intended donation of saliva.
4. No history of Hepatitis A or B.
5. No ear-nose-throat complaints.
6. Not undergoing dental treatment
7. Not wearing dentures.
8. No history of diabetes or hypoglycaemia.
9. No history of allergies including food allergies.
10. Not pregnant, planning to become pregnant in the near future or breast-feeding.
11. Not on medication such as lipid-lowering drugs, hormonal-replacement therapy, and evidence of hepatic dysfunction or supplements other than contraceptives.
12. No recent history of alcohol or drug abuse or other medical condition.
13. No prior history of any cancer. Participants with family history of cancer however, can be included.
14. No previous irradiation to head and neck region.
15. Fair to good oral hygiene (full-mouth clinical examinations were performed by certified dentists).

| Parameter | Age between 20 to 30 years of age | Above 50 years of age |
| --- | --- | --- |
| Number of participant | 10 | 10 |
| Mean age | 26 | 61 |
| Gender (M:F) | 8:2 | 4:6 |

**(b) High-risk individual**

1. Must have a good general state of health and above 50 years of age.
2. No fever or signs and symptoms suggesting active infection/illness on the day of saliva donation.
3. No mouth ulcers, inflammation of the gums, halitosis, any mouth infection, dry mouth or sensitive teeth on the day of intended donation of saliva.
4. No history of Hepatitis A or B.
5. No ear-nose-throat complaints.
6. Not undergoing dental treatment
7. Not wearing dentures.
8. No history of diabetes or hypoglycaemia.
9. No history of allergies including food allergies.
10. Not pregnant, planning to become pregnant in the near future or breast-feeding.
11. Not on medication such as lipid-lowering drugs, hormonal-replacement therapy, and evidence of hepatic dysfunction or supplements other than contraceptives.
12. No recent history of alcohol or drug abuse or other medical condition.
13. No prior history of any cancer. Participants with family history of cancer however, can be included.
14. No previous irradiation to head and neck region.
15. Suffers from gingivitis or periodontitis (full-mouth clinical examinations were performed by certified dentists).

| Parameter | Above 50 years of age |
| --- | --- |
| Number of participant | 11 |
| Mean age | 59 |
| Gender (M:F) | 8:3 |
