# Supplementary material for: The Performance of an Oral Microbiome Biomarker Panel in Predicting Oral Cavity and Oropharyngeal Cancers
Source: Front Cell Infect Microbiol. 2018 Aug 3;8:267. doi: 10.3389/fcimb.2018.00267 (PMC6085444; doi:10.3389/fcimb.2018.00267)
Supplement: Supplementary file 2 [file Table_2.DOCX]

**Supplementary Table 2. Kruskal-Wallis rank test: Oral microbiome profile comparison between individual categories of interest.**


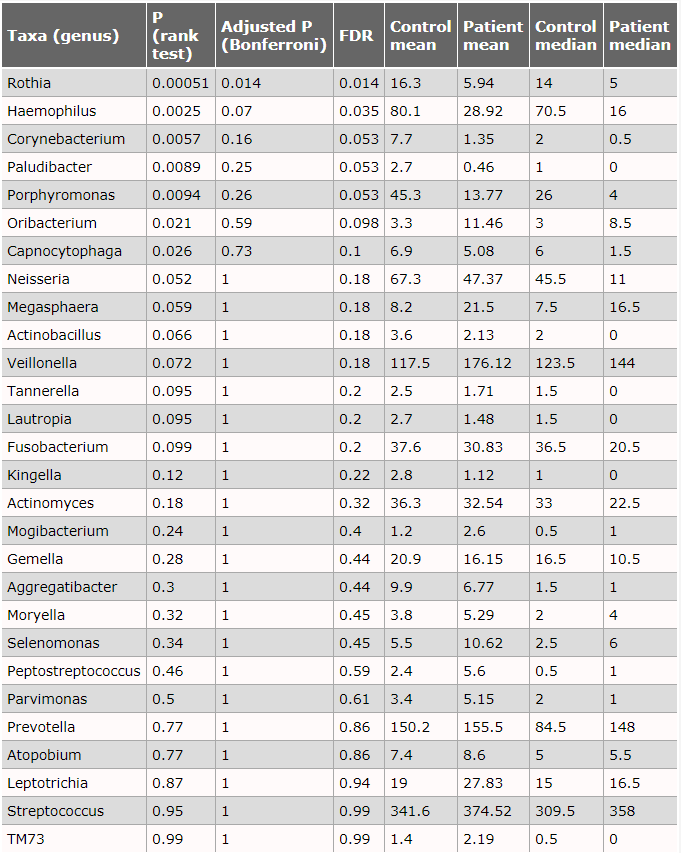


(a) Oral microbiome profile comparison between normal healthy controls (n = 10, above 50 years of age) and oral cavity and oropharyngeal cancer patients (n = 52) at genus level.


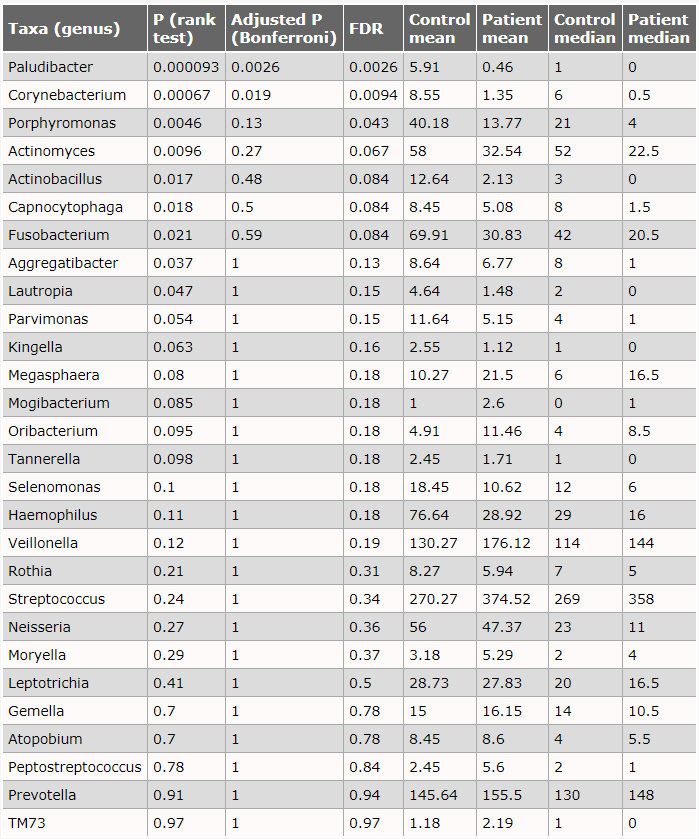


(b) Oral microbiome profile comparison between high-risk individuals (n = 11) and oral cavity and oropharyngeal cancer patients (n = 52) at genus level.


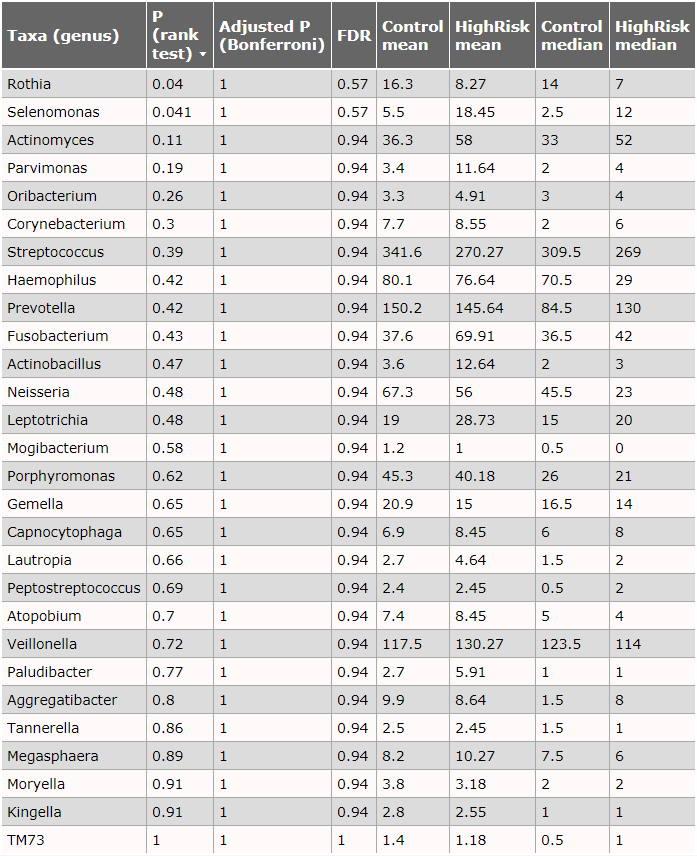


(c) Oral microbiome profile comparison between normal healthy controls (n = 10, above 50 years of age) and high-risk individuals (n = 11) at genus level.


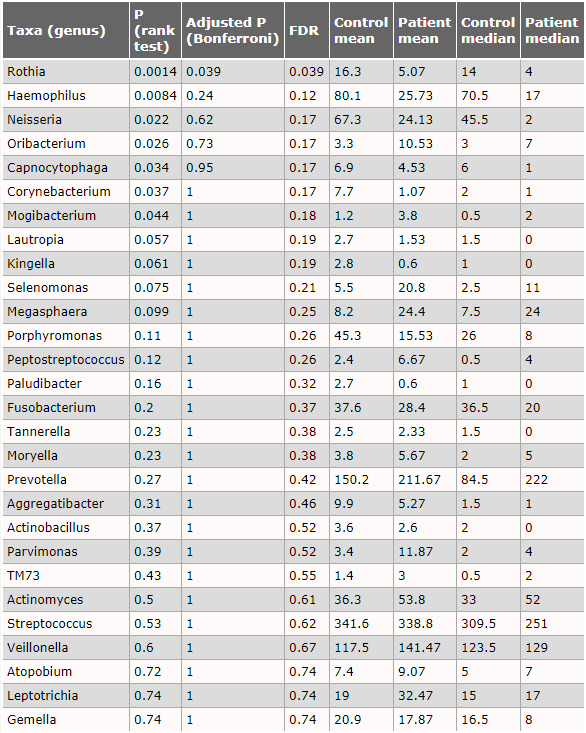


(d) Oral microbiome profile comparison between normal healthy controls (n = 10, above 50 years of age) and oral cavity cancer patients (n = 15) at genus level.


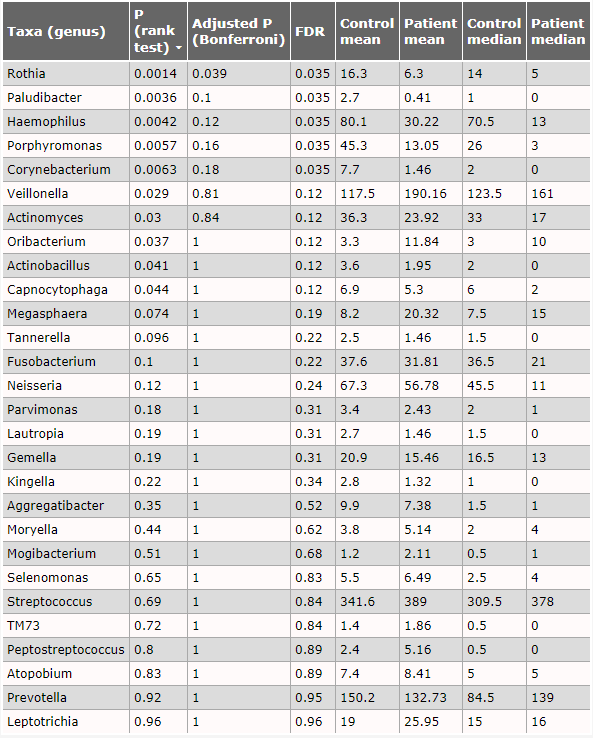


(e) Oral microbiome profile comparison between normal healthy controls (n = 10, above 50 years of age) and oropharyngeal cancer patients (n = 37) at genus level.


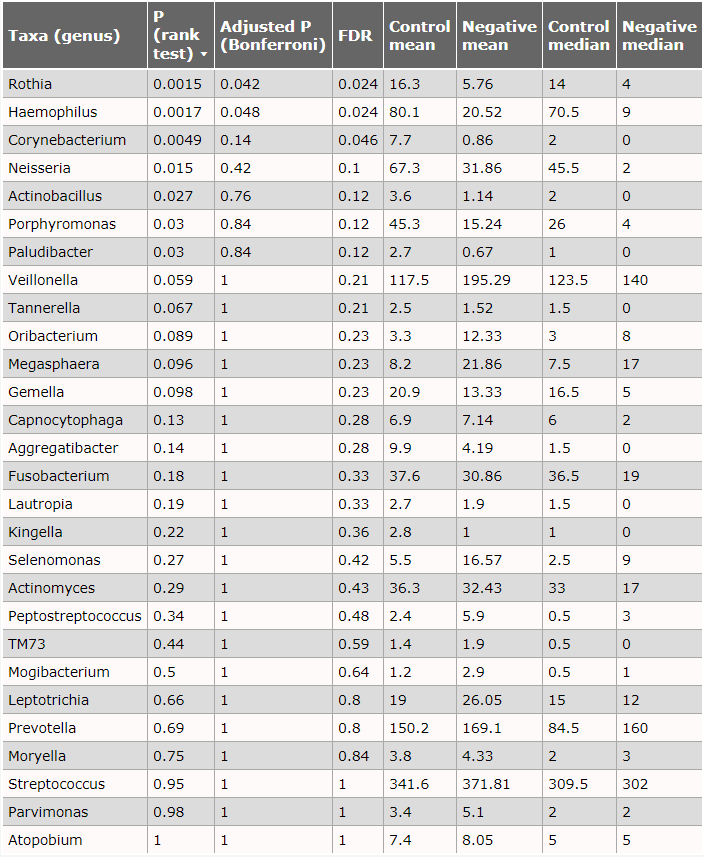


(f) Oral microbiome profile comparison between normal healthy controls (n = 10, above 50 years of age) and HPV-negative oral cavity and oropharyngeal cancer patients (n = 21) at genus level.


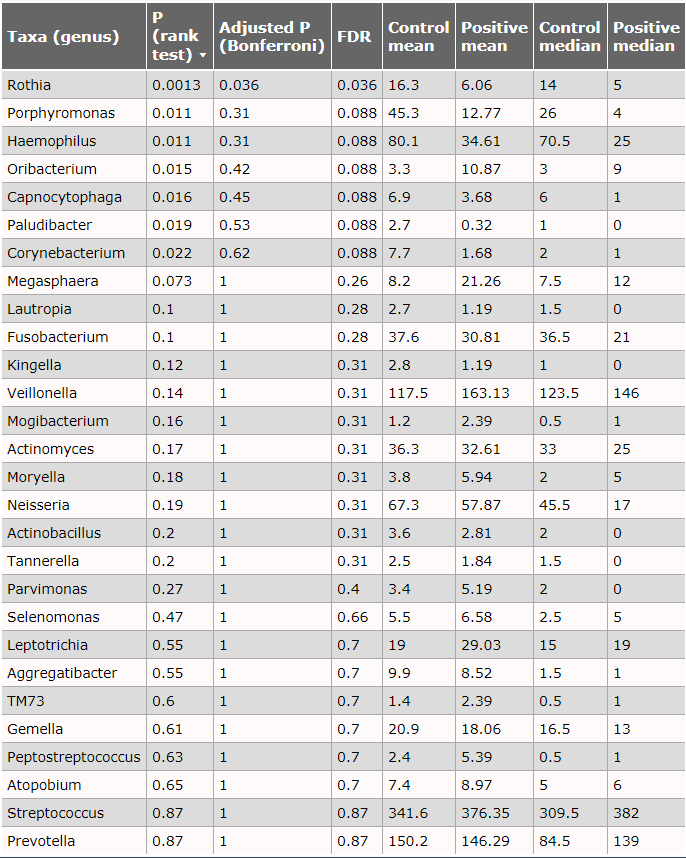


(g) Oral microbiome profile comparison between normal healthy controls (n = 10, above 50 years of age) and HPV-positive oral cavity and oropharyngeal cancer patients (n = 31) at genus level.


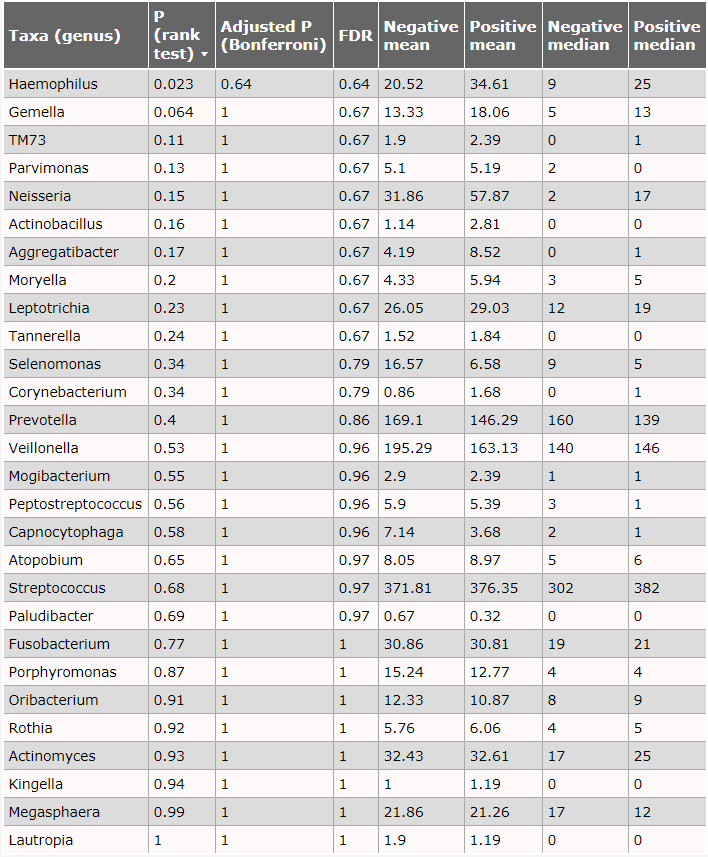


(h) Oral microbiome profile comparison between HPV-negative oral cavity and oropharyngeal cancer patients (n = 21) and HPV-positive oral cavity and oropharyngeal cancer patients (n = 31) at genus level.


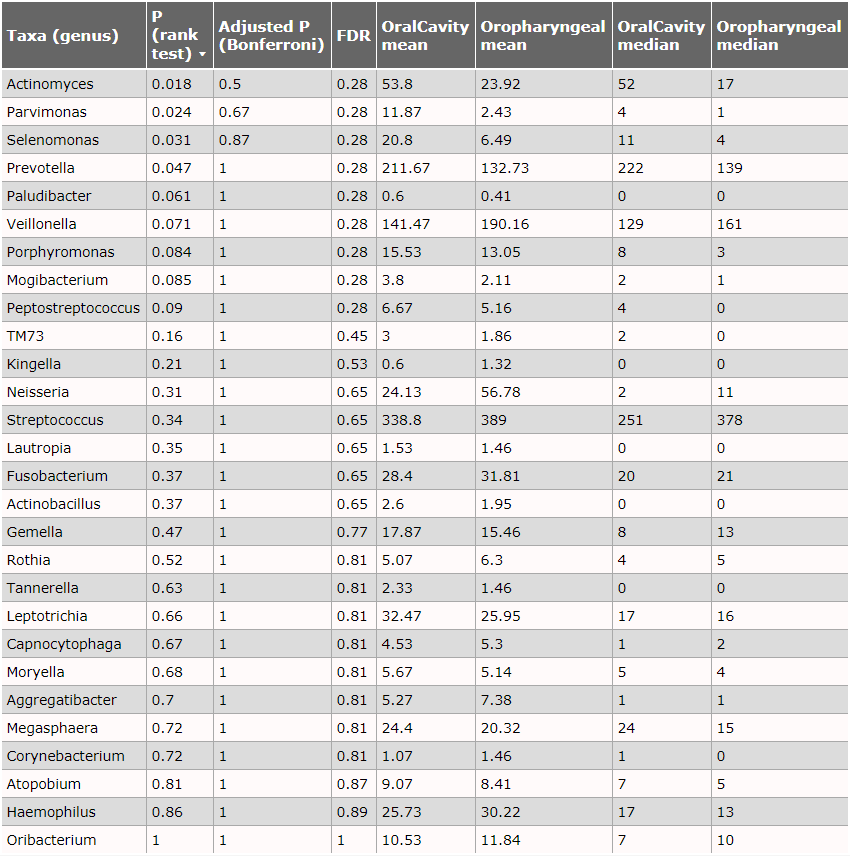


(i) Oral microbiome profile comparison between oral cavity cancer patients (n = 15) and oropharyngeal cancer patients (n = 37) at genus level.
